# Taxonomical and Functional Assessment of the Endometrial Microbiota in A Context of Recurrent Reproductive Failure: A Case Report

**DOI:** 10.3390/pathogens8040205

**Published:** 2019-10-24

**Authors:** Iolanda Garcia-Grau, David Perez-Villaroya, Davide Bau, Marta Gonzalez-Monfort, Felipe Vilella, Inmaculada Moreno, Carlos Simon

**Affiliations:** 1 Igenomix Foundation - INCLIVA Biomedical Research Institute, 46980 Valencia, Spain; iolanda.garcia@igenomix.com (I.G.-G.); marta.gonzalez@igenomix.com (M.G.-M.); felipe.vilella@igenomix.com (F.V.); 2 Department of Pediatrics, Obstetrics and Gynecology, School of Medicine, University of Valencia, 46010 Valencia, Spain; 3 Bioinformatics Department, Igenomix R&D, 46980 Valencia, Spain; david.perez@igenomix.com (D.P.-V.); davide.bau@igenomix.com (D.B.); 4 Research Department, Igenomix R&D, 46980 Valencia, Spain; 5 Department of Obstetrics and Gynecology, Baylor College of Medicine, Houston, TX 77030, USA; 6 BIDMC, Harvard University, Boston, MA 02138, USA

**Keywords:** reproductive failure, clinical miscarriage, ectopic pregnancy, endometrial microbiota, *Gardnerella vaginalis*, 16S rRNA gene sequencing, whole metagenome sequencing, metronidazole resistance, biofilm formation

## Abstract

Investigation of the microbial community in the female reproductive tract has revealed that the replacement of a community dominated by *Lactobacillus* with pathogenic bacteria may be associated with implantation failure or early spontaneous abortion in patients undergoing assisted reproductive technology (ART) treatment. Herein we describe taxonomically and functionally the endometrial microbiome of an infertile patient with repeated reproductive failures (involving an ectopic pregnancy and two clinical miscarriages). The microbiological follow-up is presented over 18-month in which the microbiota was evaluated in six endometrial fluid samples. The microbial profile of 16S rRNA gene sequencing showed a persistent infection with *Gardnerella* and other bacterial taxa such as *Atopobium* and *Bifidobacterium*. In addition, taxonomic and functional analysis by whole metagenome sequencing in the endometrial fluid sample collected before one clinical miscarriage suggested the presence of multiple *Gardnerella vaginalis* clades with a greater abundance of clade 4, usually associated with metronidazole resistance. These results revealed a persistent *G. vaginalis* endometrial colonization presenting genetic features consistent with antimicrobial resistance, biofilm formation, and other virulence factors, which could be related to the reproductive failure observed.

## 1. Introduction

The upper genital tract houses microbiota that may influence reproductive health. Culture-based studies link the isolation of *Lactobacillus* spp. from the embryo transfer catheter with a good prognosis for in vitro fertilization (IVF) [1,2,3]. Yet, other bacteria are more likely to promote inflammation and immune activation, as well as reproductive impairment. In particular, the isolation of endometrial pathogens such as *Escherichia coli*, *Streptococcus*, *Haemophilus* spp., etc. is associated with a significant decrease in implantation and pregnancy rates [1,2,4,5,6]. 

A common pathogenic bacterium infecting the reproductive tract is *Gardnerella vaginalis* [7,8,9]. The abundance of *Gardnerella* in the endometrium of patients undergoing IVF, as determined by 16S ribosomal RNA (16S rRNA) gene sequencing, is associated with clinical miscarriage or no pregnancy [10]. Furthermore, *G. vaginalis* infection follows a variable clinical course that is prone to produce recurrent episodes due to the formation of biofilms, which may resist standard therapy with metronidazole [11]. The potential for adverse reproductive consequences from this pathogen underscores the need for further research to define these links.

**Figure 1:**
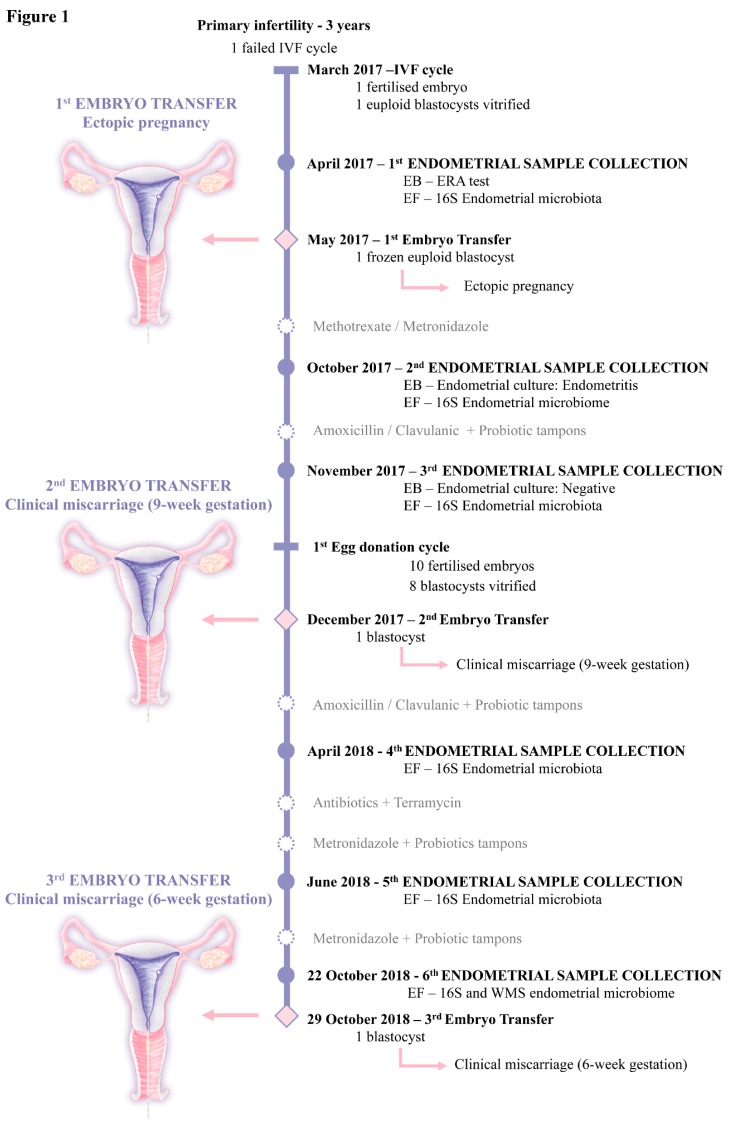
Flow chart of the clinical evolution of the patient undergoing an assisted reproductive treatment (ART) cycle with repeated reproductive failures (one ectopic pregnancy and two spontaneous abortions). EB: endometrial biopsy, EF: endometrial fluid, ERA: endometrial receptivity analysis, ICSI: intracytoplasmic sperm injection, IVF: in vitro fertilization, ET: embryo transfer, 16S: 16S ribosomal RNA gene sequencing, WMS: whole metagenome sequencing.

Here, we report the microbiological follow-up of a clinical case with repeated reproductive failures in the presence of *G. vaginalis*. 16S rRNA sequencing was used to evaluate dysbiosis of the endometrial microbiota with *Gardnerella* and other bacterial pathogens. In addition, we included whole metagenome sequencing (WMS) to further understand the proportion of *G. vaginalis*, its functionality, antibiotic resistance, and impact on reproductive health in greater depth (Appendix A Supplemental data and Figure 1).

## 2. Case Presentation

In March 2017, a 37-year-old woman with primary infertility of three years and a history of one IVF failure attended the clinic to undergo an assisted reproductive treatment (ART). Following intracytoplasmic sperm injection, two metaphase-II oocytes were recovered, but only one embryo reached the blastocyst stage. Genetic pre-implantation testing for aneuploidy identified this embryo as euploid and therefore the blastocyst was cryopreserved for transfer. 

In April 2017, the patient was included in a biomedical research study approved by the IRB of Instituto Valenciano de Infertilidad (1606-IGX-044-CS), in which the endometrial microbiota was analyzed blindly from the endometrial fluid (EF) collected at the time of endometrial receptivity analysis to study the personalized window of implantation. As this was an observational study, the patient was prepared for a personalized embryo transfer (ET) in the subsequent cycle, blinded to the microbiota results.

**Figure 2:**
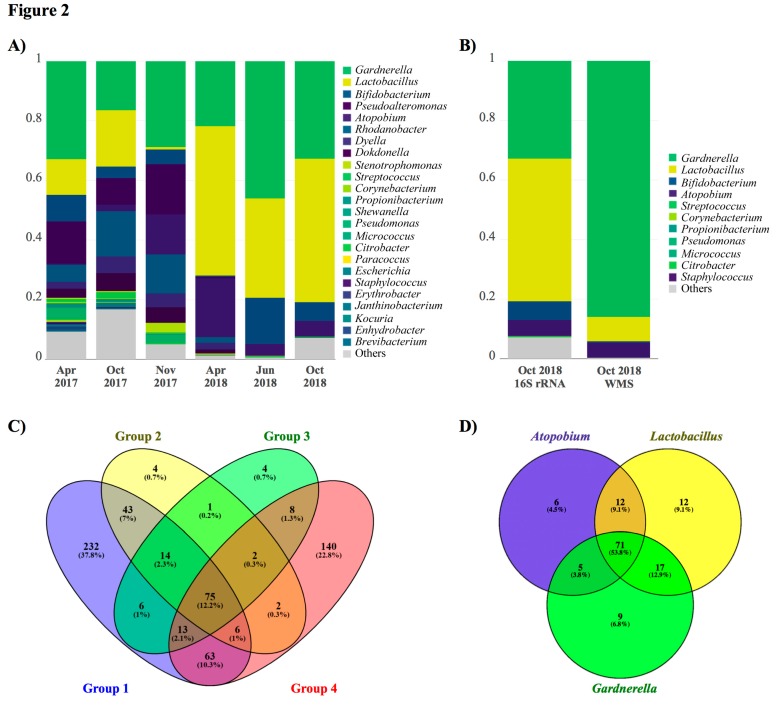
Taxonomic and functional analysis of the endometrial microbiota from an IVF patient with repeated reproductive failures. (**A**) Endometrial microbiota profile of six endometrial fluid samples assessed by 16S ribosomal RNA gene sequencing. (**B**) Comparison between 16S rRNA and whole-metagenome sequencing (WMS) in the sample taken seven days before the last ET (October 2018). (**C**) Venn diagram showing the number of genes associated with the four *G. vaginalis* clades. (**D**) Venn diagram summarizing the KEGG functional categories found in *Gardnerella*, *Atopobium,* and *Lactobacillus*.

In May 2017, one euploid blastocyst was transferred, resulting in an ectopic pregnancy. 16S rRNA sequencing in the EF collected before ET showed a bacterial population with *Gardnerella* (32.8%), *Pseudoalteromonas* (14.2%), *Bifidobacterium* (8.8%), and *Rhodanobacter* (5.8%), with only 12.1% *Lactobacillus* (Figure 2A).

The patient was treated with methotrexate (1 mg/kg, intramuscular) for the ectopic pregnancy and metronidazole (single vaginal dose of 500 mg). In October 2017, a biopsy and EF sample were obtained to analyze the presence of pathogenic bacteria by microbial culture and sequencing, respectively. 16S rRNA sequencing revealed an altered microbial composition: *Lactobacillus* (18.8%), *Gardnerella* (16.5%), *Rhodanobacter* (15.2%), and *Pseudoalteromonas* (9.0%) (Figure 2A). The analysis of the endometrial tissue confirmed the presence of endometritis with abundant Gram negative bacillus and positive culture for *Escherichia coli*. The patient was prescribed a course of amoxicillin/clavulanic acid (1,000/62.5 mg twice daily for seven days), followed by *Lactobacillus* probiotic tampons (three tampons daily for three days during menses). 

One month later, in November 2017, 16S rRNA sequencing from the endometrium and EF revealed the microbiota composition as *Gardnerella* (28.8%), *Pseudoalteromonas* (16.8%), *Atopobium* (13.4%), *Rhodanobacter* (13.0%), and *Lactobacillus* (0.8%) (Figure 2A). However, microbial culture was negative. The patient decided to continue with a new ART cycle following the standard of care. The patient exhibited low ovarian reserve, and she elected to initiate an ovum donation cycle. Of 12 metaphase-II donor oocytes, 10 were fertilized and eight embryos reached the blastocyst stage. ET with one blastocyst, performed in December 2017, resulted in a clinical pregnancy that ended in clinical miscarriage at the 9th week of gestation. 

In the following months, several antibiotics (amoxicillin/clavulanic acid (500/125 mg every 8 h for seven days) and metronidazole (500 mg twice daily for seven days)) together with *Lactobacillus* vaginal probiotic tampons (three daily for three days) were administered to the patient. After each treatment, an EF sample was taken to evaluate changes in the endometrial microbiota composition (April and June 2018). 16 rRNA detected a persistent colonization of *Gardnerella* (21.8% and 46.1%, in respective time points), *Atopobium* (19.7% and 4.1%) and *Bifidobacterium* (0.3% and 15.5%) with suboptimal levels of *Lactobacillus* (50.2% and 33. 4%) (Figure 2A). 

The patient was again treated with metronidazole and probiotic tampons. An EF was obtained in the same cycle as a third ET (using ovum donation) that was performed in October 2018. Sequencing revealed the presence of *Lactobacillus* (48.1%), *Gardnerella* (32.8%), *Bifidobacterium* (6.2%), and *Atopobium* (5.4%) (Figure 2A). As in the previous attempts, pregnancy was achieved and the gestational sac was visualized at the 5th week, but clinical miscarriage occurred at the 6th week of gestation.

We performed a more complete taxonomic and functional analysis by WMS in the last EF sample obtained seven days before the ET that resulted in this 3rd reproductive failure. Taxonomic analysis using WMS verified the dysbiotic profile dominated by *Gardnerella* (86.0%), *Lactobacillus* (8.2%) and *Atopobium* (5.1%) (Figure 2B). While these microorganisms were in agreement with those detected by 16S rRNA sequencing, the proportions differed - maybe due to differences in the experimental protocols and bioinformatic pipelines applied to each sequencing method. The taxonomic analysis by WMS also provided information on functional taxa at the species level, confirming that the most abundant bacterium in the sample was *G. vaginalis*. 

Comparative genomic studies reveal that *G. vaginalis* has a population of four clades/ecotypes with distinct genomic properties that may confer different ecological functions [12], and clades 3 and 4 are associated with metronidazole resistance [13]. Here, sequences obtained from *Gardnerella* were compared with the genomes of a panel of 17 *G. vaginalis* strains belonging to different clades [12]. A total of 613 significant sequence alignments were associated with genes from these strains, most of them matching with clade 1 (n = 452) and clade 4 (n = 309), with 232 and 140 unique genes of these two ecotypes, respectively. Conversely, clade 2 and clade 3 were annotated to a lesser extent, with only four genes specific to each of these two clades (Figure 2C). 

To study the particular abundance of each ecotype, the genomic sequences obtained were searched for the presence of clade-specific genes, following the work by Balashov and collaborators [14]. Analysis of the genetic markers showed the concomitant presence of multiple *G. vaginalis* clades, clade 4 was the most represented, followed by clade 3 and clade 1. Specifically, for clade 4 the chloride transporter CIC family and allantoate amidohydrolase were detected with 192 and 306 reads per kilobase million (RPKM), respectively. For clade 3, thioredoxin and α-β hydrolase fold proteins were interrogated, with 256 RPKM detected for the thioredoxin gene only, and for clade 1, α-L-fucosidase and galactokinase genes were detected with 104 and 114 RPKM, respectively. No sequences belonging to the α-β hydrolase fold protein encoding gene (clade 3) or the clade 2 genes (hypothetical protein and cellulosome anchoring protein) were found in the sample. The predicted clades in the sample were confirmed by multiplex qPCR for clade-specific genes [14], showing positive detection of clades 1 and 4.

**Figure 3:**
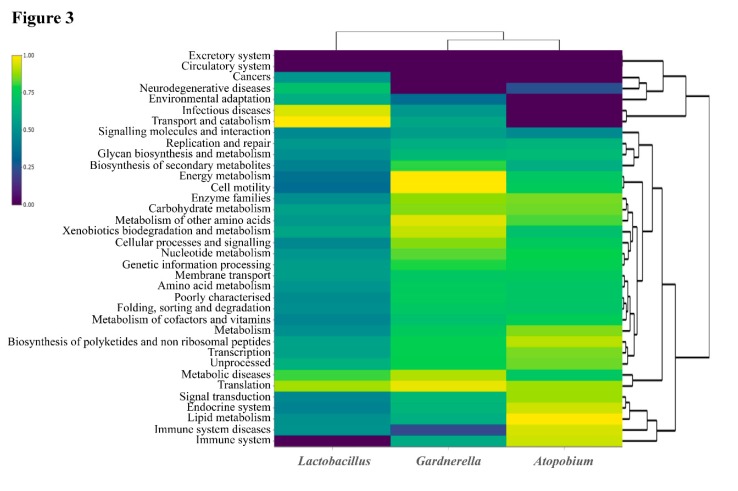
Heat map representing the most abundant KEGG categories associated with the genera *Gardnerella*, *Atopobium* and *Lactobacillus* in the endometrial fluid sample collected seven days before the embryo transfer (ET) that resulted in a clinical miscarriage (October 2018).

Furthermore, the functional metagenomics analysis by WMS was performed using the Kyoto Encyclopedia of Genes and Genomes (KEGG) database. Most functional categories detected belonged to the genera *Gardnerella*, *Lactobacillus* and *Atopobium* (representing 66%, 18%, and 14% of detected reads, respectively) (Figure 2D). Nine categories were exclusive of *Gardnerella*, comprising pathways related to metabolism (fatty acids biosynthesis, sulfur metabolism, and inositol phosphate metabolism), cell motility (including bacterial chemotaxis and motility proteins), signaling processes (such as sporulation and bacterial toxins) and immune system (NOD-like receptor signaling pathway). In contrast, 12 functional categories were exclusive of Lactobacillus, most of which were related to general functions such as metabolism, genetic information and signaling processes (Figure 3, Appendix B Table A1). 

The sample was interrogated for the presence of genes previously described to contribute to the pathogenic potential of *G. vaginalis* [15]. Several detected genes were related to (i) toxin-antitoxin (TA) system and competitive exclusion, (ii) biofilm formation and epithelial adhesion, and (iii) virulence factors including cytotoxicity, antimicrobial resistance, iron acquisition, and mucin degradation, among others (Table 1).

## 3. Discussion

Numerous studies report the inconsistent efficacy of routine *G. vaginalis* therapies. The current options do not successfully manage this microbial condition. Metronidazole is the first-line drug of choice because it is also effective against other anaerobes. However, treatment efficacy is limited to the short term, and symptoms usually return in 20% of patients with bacterial vaginosis within one month [16], and 58% within one year [17]. Antimicrobial resistance could be due to the fact that *G. vaginalis* and *Atopobium vaginae* strains present a variable susceptibility for metronidazole, and some even show intrinsic resistance [18]. Indeed, metronidazole-resistant strains of these bacteria are detected in 100% and 75% of women with recurrent bacterial vaginosis after antibiotic therapy [19]. For this reason, alternative therapies under consideration include other compounds (amoxicillin/clavulanic acid or clindamycin), routes of administration and antibiotic regimen (dose and duration) [20]. Several studies also evaluated the clinical and microbiological efficacy of probiotics to treat and/or prevent the recurrence of reproductive tract infections, denoting the value of including probiotics as part of the approach to prevent and/or treat these diseases [21]. Despite the need for additional clinical studies, probiotics were used here as an adjunct to antimicrobial treatment, resulting in a gradual increase in the percentage of *Lactobacillus* after its administration in the last three EF samples analyzed (Figure 2A). However, the main problem is the resistance of *G. vaginalis* and other pathogens to the treatment. 

Indeed, despite the antibiotic and probiotic treatments administered to the patient, the six EF samples analyzed at different time points over 18 months showed persistent *Gardnerella* infection. *G. vaginalis* is a sexually-acquired pathogen that produces infectious conditions (such as bacterial vaginosis, endometritis and/or pelvic inflammatory disease) through the establishment of a bacterial biofilm along the female reproductive tract [8]. Biofilms are formed by a dense and tight network of bacterial cells enclosed within a fibrillar exopolysaccharide matrix, which confers a strong adherence to the host tissues (and even catheters or protheses) and impedes access by metronidazole [11]. Moreover, *G. vaginalis* biofilms are usually strongly adhered to the epithelium and can be present for prolonged periods of time without causing symptoms [11], which could explain the recurrent nature of this condition and the subsequent treatment failures observed in this case. Finally, the high recurrence presented herein may be explained by possible reinfection, since a higher recurrence rate has been observed in women with a unique sex partner compared to women with a new partner [17].

Advances in next-generation sequencing have enabled differentiation of four ecotypes of *G. vaginalis* according to sequence variations [12]. After analyzing clade-specific genetic markers by WMS, we observed the concomitant presence of multiple clades in one sample, which has been identified as a risk factor in women with genital infections such as bacterial vaginosis [14]. Specifically, we detected a greater abundance of clade 4 and clade 3 in the sample collected seven days before an ET that ended up in a 6-week clinical miscarriage. Interestingly, these clades have been reported to confer intrinsic resistance to metronidazole [13]. After analysing clade-specific genetic markers, the most abundant in our sample was a unique operon of clade 4 associated with allantoin utilization as a nitrogen source under adverse conditions. This unique scavenging/foraging ability is absent in other *Gardnerella* subtypes and may represent an advantage for its survival [12]. Thus, it could be important to identify the gene differences responsible for the production of particular pathogenicity traits and clinical phenotypes, to inform appropriate measures toward preventing adverse reproductive outcomes. A diagnostic tool that evaluates the presence of *G. vaginalis* and distinguishes its clades may be useful to treat infertile patients and identify those cases that may be at a higher risk for recurrent disease. 

## 4. Conclusions

In conclusion, here we present the 18-month follow-up of a clinical case involving an ectopic pregnancy and two early clinical miscarriages, in whom the endometrial microbiota was evaluated in a total of six endometrial samples. Although other factors cannot be completely ruled out, our 16S rRNA sequencing results reveal a persistent *G. vaginalis* colonization, which could be related to the reproductive failure since embryonic factor was considered unlikely. Furthermore, WMS of the last EF sample suggested the presence of multiple *G. vaginalis* clades with a greater abundance of clade 4, which may be related to metronidazole resistance and endometrial infection recurrence, together with virulence genes involved in TA system, biofilm formation, and antibiotic resistance. The implications of this case report may contribute to medical awareness of the potential impact of microbial pathogens in the management of infertility. Likewise, it leads us to propose new diagnostic tools and personalized therapeutic procedures through molecular microbiological evaluation.

## Figures and Tables

**Table 1:** *G. vaginalis* pathogenic-associated genes detected in the sample collected in the same cycle of the second clinical miscarriage (Yeoman et al., 2010).

**ADAPTATION TO ENVIRONMENT**	
*Mobile elements and horizontal gene transfer:*	Recombinase (RecA); Transposase IS3509a; HK97 family phage major capsid protein; Site-specific recombinase phage integrase family; Helicase UvrD/REP; Phage related protein.
*Competence:*	Putative competence-damage inducible protein (CinA); ABC-type antimicrobial peptide transporter permease component; Glycoside hydrolase (GH) family; Lysozyme; Penicillin-binding protein; Fic-family protein; M13 family peptidase; ATP-binding subunit of Clp protease.
*Toxin-antitoxin system:*	RelB toxin/antitoxin family; Antitoxin/DNA-binding transcriptional repressor DinJ.
**VIRULENCE**	
*Biofilm formation and exopolysaccharide formation:*	Glycosyltransferase (GT) type II; Sortases; LPxTG domain; Actinobacterial surface anchored protein domain.
*Epithelial adhesion:*	Type I fimbrial major subunit precursor; Pilus assembly protein (PilY1); Tfp pilus.
*Antimicrobial resistance:*	Efflux transporter; ABC-type multidrug transport system; ABC-type bacteriocin/lantibiotic; Multidrug resistance transporter EmrB/QacA; Bleomycin hydrolase; SalY-type ABC-antimicrobial peptide transport system; Cadmium resistance transporter CadD family protein.
*Mucin degradation:*	Alpha-mannosidase; Beta-galactosidase.
*Cytotoxicity and hemolysis:*	Hemolysin-like protein.
*Iron intake and utilisation:*	FTR1-family iron permease; FtsK/SpoIIIE family protein.
*Other virulence factors:*	G-related albumin-binding (GA) modules; Virulence-associated E family; Oxygen-insensitive NAPDH nitroreductase (RdxA).

## Appendix A

**SUPPLEMENTAL DATA: METHODS**

**Sample collection**

Endometrial fluid (EF) samples were obtained from the same patient by transcervical aspiration with a double lumen embryo transfer catheter as previously described [22]. Collection of endometrial fluid samples was approved by the local ethical committee at Instituto Valenciano de Infertilidad (Federalwide Assurance number: FWA00027749) with protocol number 1606-IGX-044-CS. The patient provided signed informed consent for the aspiration of the endometrial fluid samples and the subsequent publication of her case.

EF specimens were collected in sterile tubes containing 50 μL of RNA*later* solution (Thermo Fisher Scientific, Waltham, MA) following the manufacturer’s instructions and stored at −80°C until use. EF aspiration before IVF embryo transfer is a safe method for assessing endometrial samples in the embryo transfer cycle without affecting implantation rates [23].

**DNA extraction**

Total DNA was isolated by performing a pre-digestion step with lysozyme, lysostaphin, and mutanolysin to degrade the cell walls of bacteria, followed by extraction with QIAamp DNA Blood Mini kit (Qiagen, Hilden, Germany) following the manufacturer’s instructions. Genomic DNA was quantified using Tape Station (Agilent technologies, Waldbronn, Germany) and subjected to preamplification and 16S rRNA sequencing for the identification of endometrial microbiota represented in EF.

**16S ribosomal RNA sequencing**

Microbiota profiles were obtained by 16S rRNA gene sequencing using the Ion 16S metagenomics kit (ThermoFisher Scientific, Waltham, MA). This kit includes two primer sets (V2-4-8 and V3-6, 7-9) that selectively amplify the corresponding hypervariable regions of the 16S ribosomal subunit. Amplified fragments were sequenced on the Ion S5 XL system (ThermoFisher Scientific) and results were analysed using the QIIME 2.0 package (https://qiime2.org) and RDP classifier 2.2 for taxonomic assignment. Positive controls of *Escherichia coli* DNA along with blank controls were included in the assays to detect any potential contamination from reagents.

**Whole metagenome sequencing**

Endometrial microbiome functional composition was assessed by whole metagenome sequencing (WMS) using the Nextera DNA Flex Library Preparation kit (Illumina, San Diego, CA) following the manufacturer’s instructions. Libraries were then sequenced on the NextSeq 500 system (Illumina, San Diego, CA). Reads generated by Illumina sequencing platform were quality-trimmed and length-filtered using PRINSEQ [24]. Paired-end reads were merged using Fast Length Adjustment of Short reads (FLASh) software tool [25] and, finally, host-reads were removed using Burrows-Wheeler Aligner (BWA) mapped against human genome reference [26].

Functional and taxonomical profiling was obtained using the co-assembly procedure from SqueezeMeta automatic pipeline [27]. This method pools reads from all samples and a single shared assembly is defined. ORF prediction and annotation are retrieved by homology searching on co-assembly contigs using KEGG [28] reference databases. Gene abundance was estimated mapping individual reads to the reference co-assembly contigs annotation. Finally, resulting SqueezeMeta outputs were processed using R statistical software [29] for statistical description and graphical representation of the sample’s taxonomical and functional profile.

**Quantitative polymerase chain reaction**

TaqMan quantitative polymerase chain reactions (qPCRs) targeting clade-specific genes for *Gardnerella* were performed as previously reported [14]. Primers for four clade-specific genes (clade 1:α-L-fucosidase, clade 2: hypothetical protein, clade 3: thioredoxin and clade 4: chloride transporter CIC family) and probes coupled to different fluorophores (FAM, TAMRA, ROX, and Cy5, respectively) were purchased from Integrated DNA Technologies (IDT, Belgium). Multiplex TaqMan qPCRs were performed in 20-µL reactions containing 1× PrimeTime Gene Expression Master Mix protocol (IDT, Belgiun), 800 nM each DNA primer, 100 nM each TaqMan probe and 3.2 µL DNA. Cycling parameters were 50 °C for 2 min for uracyl-N-glycosidase (UNG) treatment and 95 °C for 3 min for initial denaturation, followed by 40 cycles of denaturation at 95 °C for 15 s and annealing plus extension at 60 °C for 1 min with fluorescence acquisition at the end of each cycle. QuantStudio 5 Real-Time PCR System (Thermo Fisher Scientific) was used for qPCRs. As a positive control, DNA from *G. vaginalis* (type strain ATCC 14018) was included in the PCR at concentrations ranging from 0 to 10^6^ genome copies.

**Data availability**

The genetic sequences generated in this study are deposited in the SRA database under the accession number PRJNA545633.

## Appendix B

**Table A1:** KEGG categories (Level 2-Level 3) exclusively associated with the genera *Gardnerella*, *Atopobium* and *Lactobacillus*. Numbers represent the reads per kilobase million (RPKM) associated with each function/taxon.

**9 categories included exclusively in *Gardnerella***	**RPKM**
Lipid metabolism-Fatty acid biosynthesis	729.54
Cell motility-Bacterial chemotaxis	559.63
Energy metabolism-Sulfur metabolism	303.44
Cellular processes and signalling-Sporulation	225.36
Signaling molecules and interaction-Bacterial toxins	207.02
Cell motility-Bacterial motility proteins	155.71
Environmental adaptation-Circadian rhythm - Plant	137.72
Carbohydrate metabolism-Inositol phosphate metabolism	132.10
Immune system-NOD-like receptor signalling pathway	115.83
**6 categories included exclusively in *Atopobium***	**RPKM**
Metabolism-Nucleotide metabolism	92.61
Metabolism of cofactors and vitamins-Folate biosynthesis	68.42
Cellular processes and signalling-Cell motility and secretion	55.19
Immune system-RIG-I-like receptor signalling pathway	38.13
Metabolism of other amino acids-Beta-alanine metabolism	34.60
Neurodegenerative diseases-Alzheimer’s disease	10.19
**12 categories included exclusively in *Lactobacillus***	**RPKM**
Carbohydrate metabolism-C5-branched dibasic acid metabolism	34.35
Genetic information processing-Transcription related proteins	33.44
Metabolic diseases-Type I diabetes mellitus	25.47
Carbohydrate metabolism-Pentose and glucuronate interconversions	24.87
Glycan biosynthesis and metabolism-Lipopolysaccharide biosynthesis proteins	24.49
Environmental adaptation-Plant-pathogen interaction	22.52
Xenobiotics biodegradation and metabolism-Benzoate degradation via hydroxylation	16.80
Metabolism of cofactors and vitamins-Ubiquinone and other terpenoid-quinone biosynthesis	15.18
Carbohydrate metabolism-Citrate cycle (TCA cycle)	12.50
Metabolic diseases-Type II diabetes mellitus	10.66
Cellular processes and signalling-Pores ion channels	6.16
Metabolism-Amino acid metabolism	4.21
